# Overcoming Recurrent Miscarriages in a 35-Year-Old Female With Thrombophilia

**DOI:** 10.7759/cureus.62870

**Published:** 2024-06-21

**Authors:** Mira Hristova

**Affiliations:** 1 Department of Obstetrics and Gynecology, Multiprofile Hospital for Active Treatment (MHAT) "NiaMed" OOD, Stara Zagora, BGR

**Keywords:** thrombophilias, recurrent pregnancy loss, pregnancy complications, pai-1 (4g/5g), nk cells, mthfr (ala222val)

## Abstract

Thrombophilias, which include both hereditary and acquired illnesses, are a range of abnormalities that make persons more prone to developing thromboembolism. Thrombophilic conditions carry significant dangers during pregnancy, such as miscarriage in early pregnancy, intrauterine growth restriction, abruptio placenta, and preeclampsia. According to compiled statistics, an average of 15%-20% of pregnancies end in miscarriage. While the risk of miscarriage in a first pregnancy is 11%, this risk increases to between 13% and 17% in subsequent pregnancies, and after the third miscarriage, it reaches 38%.

This research article presents a detailed case report that focuses on a patient who has experienced three previous failed pregnancies. The patient's genetic analysis indicates that she has two copies of a mutated version of the methylenetetrahydrofolate reductase (*MTHFR*) gene (Ala222Val) and a variation in the plasminogen activator inhibitor 1 (*PAI-1*) gene known as 4G/5G. In addition, an evaluation of immunological characteristics revealed increased amounts of natural killer (NK) cells with enhanced activity, along with the identification of embryotoxins in a blood test that suppress embryotoxicity in a blood test, assisted by DNA isolation and real-time polymerase chain reaction (PCR) DNA analysis.

## Introduction

Thrombophilia is a complex medical disorder that can be either present at birth or acquired later in life. Congenital thrombophilias include genetic mutations such as factor V Leiden and prothrombin gene mutation (G20210A). Acquired thrombophilias, on the other hand, can develop due to factors such as antiphospholipid syndrome and prolonged immobility [1,2].

It is characterized by an elevated likelihood of developing blood clots, which can block blood vessels and pose significant hazards to the health of both the mother and the fetus [3]. During a normal pregnancy, the pregnant person's body undergoes physiological changes, including modifications in blood clotting processes. However, genetic thrombophilia may disturb these adaptive systems, leading to an increased tendency to form blood clots, often without the person's awareness. Hereditary causes primarily determine congenital thrombophilic diseases, and their symptoms often remain undetected until pregnancy. Unexplained thrombophilias contribute to 40%-50% of the etiology of recurrent pregnancy loss (RPL) in pregnancy instead of problems that occur during pregnancy [4,5].

In addition to these thrombophilic disorders, there are various variables that increase the risk of thromboembolism. Significant contributions to the condition include a high body mass index (BMI), multiple pregnancies, infections, preeclampsia, being immobile during pregnancy, and being of advanced maternal age [6]. The combination of these factors can lead to different obstetric complications, such as habitual abortions, fetal death, restricted fetal growth, placental disorders such as calcification or abruption, and hypertension disorders, resulting in conditions such as preeclampsia, eclampsia, and hemolysis, elevated liver enzymes, and low platelet count (HELLP) syndrome. These multifaceted influences highlight the intricate nature of thromboembolic pathophysiology during pregnancy and stress the significance of thorough risk assessment and treatment options [7-9].

During a normal pregnancy, the patient undergoes changes in the coagulation cascade, resulting in significant variations in several coagulation factors. More specifically, there is a noticeable increase in the levels of factors Vc, VIIc, Xc, and von Willebrand, along with a decrease in the amounts of functional and free protein S [10]. Nevertheless, the levels of protein C and antithrombin III demonstrate negligible fluctuation during pregnancy. The reported rise in plasminogen activator inhibitor (PAI) levels is very important because it makes it harder for the mother's blood to flow and break up blood clots [5,6].

The complex changes in the coagulation profile can occur alone or together, leading to the development of a multifactorial thrombotic syndrome that disrupts pregnancy's normal progression. Among the myriad scenarios encountered, frequently observed deficiencies are in antithrombin, protein S, and protein C, highlighting their crucial roles in maintaining the balance of blood clotting during pregnancy [10].

## Case presentation

This study examines a clinical situation involving a patient, G3P0A3. A thorough blood test using DNA separation techniques and real-time polymerase chain reaction (PCR) DNA analysis methods confirmed the presence of inhibitory embryotoxins in the patient's blood, two copies of a mutated allele connected to the Ala222Val polymorphism in the methylenetetrahydrofolate reductase (*MTHFR*) gene. The 4G/5G genotype identifies a gene variant associated with the *PAI-1* gene in the patient. In addition, in line with these genetic discoveries, the patient demonstrates increased amounts of natural killer (NK) cells, which show enhanced activity.

The patient's three previous spontaneous abortions occurred in the first trimester of pregnancy. She reports no issues with conceiving and has not undergone any in vitro procedures. The patient came for a consultation after her third spontaneous abortion. Her last menstrual period was on September 20, 2019. After conducting genetic tests, the following findings were discovered. An in-depth examination determined that the patient is homozygous for the mutant allele of *MTHFR* (Ala222Val) and *PAI-1* (4G/5G). Surprisingly, she had high amounts of NK cells that were more active, indicating a potentially harmful effect on the embryo and a greater activation of trophoblast lymphocytes. The treatment strategy, which included low-molecular-weight heparin, immunoglobulin supplementation, and intravenous gamma globulin therapy, resulted in the successful advancement of pregnancy and eventual delivery.

**Figure 1:**
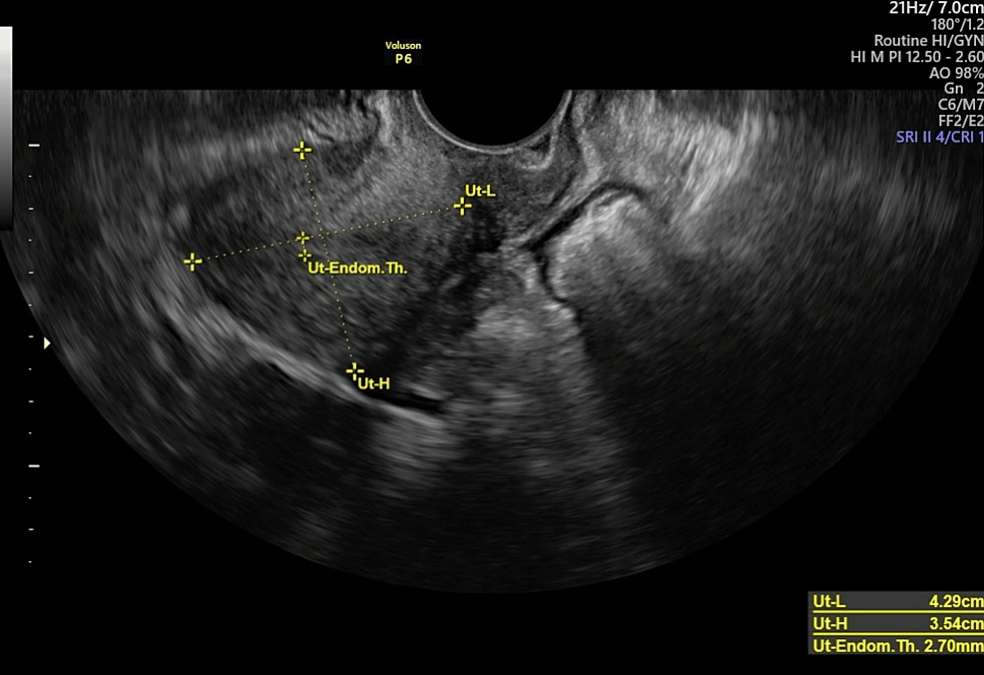
Ultrasound assessment of the uterine sizes and endometrial thickness before pregnancy

Upon examination, the patient underwent a comprehensive gynecological assessment. A Pap smear was performed, and the results were normal. Portio vaginalis cervicis uteri (PVCU) has a conical shape, indicating nulliparous status. Additionally, during palpation, the uterus showed normal attributes such as size, shape, softness, elasticity, and mobility. These clinical findings were further supported by transvaginal ultrasonography, which showed no detectable pathological changes in the adnexal structures of the uterus (Figure 1).

The diagnostic assessments revealed several findings: a homozygous state for the mutant allele *MTHFR* (Ala222Val), homozygous status for *PAI-1* (4G/5G), increased NK cell counts, the presence of inhibitory embryotoxic factors, and elevated levels of thrombocyte-leukocyte aggregates (Table 1). At the same time, laboratory tests showed the existence of several antibodies within normal levels.

**Table 1:** Thrombophilia genetic panel test SERPINE1: serpin family E member 1

Test	Result	Comment
Factor II (prothrombin) (F2 and G20210A)	G/G	Homozygote under normal allele
Factor V Leiden (F5, G1691A, Arg506Gln, and R506Q)	G/G	Homozygote under normal allele
*MTHFR* (C677T and Ala222Val)	T/T	Homozygote
*MTHFR* (A1298C and Glu429Ala)	A/A	Homozygote under normal allele
*PAI-1* (SERPINE1) (4G/5G)	5G/4G	Homozygote
Factor XII (F13A1, G103T, and Val34Leu)	G/G	Homozygote under normal allele

The treatment plan included a holistic approach, involving the use of meta-folic acid, methyl-B12, vitamin D supplements, intravenous immunoglobulin (given 2-4 days before expected ovulation), intralipid infusion (given 2-4 days after pregnancy confirmation), an aspirin protection regimen (taken every other day before and during pregnancy), and either Fraxiparine or Cloxane administration (given every other day after pregnancy confirmation). We established an ongoing surveillance system that actively evaluated the effectiveness of anticoagulant medication. This included the frequent monitoring of thrombocyte counts, D-dimer levels, and activated partial thromboplastin time (aPTT), with evaluations taking place every three weeks.

The patient's therapy started with an initial consultation at the onset of her menstrual cycle, which prompted a comprehensive gynecological assessment and the formulation of an individualized conception strategy. On the 12th day of the patient's menstrual cycle, following an ultrasound examination to measure the largest follicle in the ovary, the patient received the initial infusion of 10 g of immunoglobulin. This sparked a series of prearranged infusions, complemented by precise medication regimens adjusted according to the different stages of pregnancy. We started anticoagulant therapy with low-molecular-weight heparin and conducted frequent assessments to ensure the successful progression of a healthy and viable pregnancy. The patient's pregnancy monitoring involved the rigorous supervision of thrombolytic treatment, including regular testing of D-dimer, aPTT, and platelet levels every three weeks. We also conducted monthly ultrasound scans to monitor the fetus's development rate. Table 2 presents the aPTT, D-dimer, and platelet values for a duration of three weeks, commencing in early pregnancy and concluding at 39 weeks of gestation.

**Table 2:** The activated partial thromboplastin time (aPTT), D-dimer, and platelet values

Gestational week	aPTT (seconds)	D-dimer (μg/mL)	Platelets (×10^9^/L)
7	30	0.6	250
10	31	0.7	240
13	32	0.8	230
16	30	0.9	220
19	28	1.0	215
22	27	1.2	210
25	26	1.5	205
28	25	1.7	200
31	24	2.0	195
34	23	2.2	190
37	22	2.5	185
39	21	2.8	180

In addition to monitoring these parameters during pregnancy, regular ultrasound examinations were performed to monitor fetal growth rate, including biochemical screening at 11-13 weeks of gestation, fetal morphology between 20 and 22 weeks of gestation followed by Doppler sonography, and Doppler examination performed between 30 and 32 weeks of gestation. The prenatal ultrasound scans indicated a typical fetal growth rate. The crown rump length (CRL) of the fetus at 11-13-week gestational age was 30 mm with a normal heart rate. At 20-22-week gestational age, the fetal measurements were as follows: biparietal diameter (BPD), 50 mm; abdominal circumference, 178 mm; and femur length (FL), 35 mm. Between 30- and 32-week gestational age, the fetal measurements were as follows: BPD, 80 mm; abdominal circumference, 250 mm; and femur length, 56 mm. Performed between 20- and 22-week gestational age and between 30- and 32-week gestational age, Doppler sonographies showed normal blood flow in the uterine arteries, ductus venosus, and both umbilical arteries.

The patient underwent a caesarean section in the 39th week of gestation, after which anticoagulant therapy was discontinued. This comprehensive treatment regimen exemplifies the proficient management of a multifaceted medical condition throughout the entire pregnancy, leading to optimal outcomes for both maternal and neonatal health.

## Discussion

Throughout a normal pregnancy, complex alterations take place within the hemostatic system, carefully controlled by several physiological processes. Numerous physiological and clinical perspectives have thoroughly examined this phenomenon, which marks a significant transition toward a state of increased blood clotting. During pregnancy, there were big changes in both the coagulation and fibrinolytic pathways, which made people more likely to have thromboembolic events [11].

During pregnancy, a series of physiological events occur that affect the delicate balance of blood clotting and clot breakdown mechanisms in the mother's body. Several coagulation factors, including factors VII, VIII, X, and XII, had significantly increased, as had fibrinogen levels. On the other hand, elements of the anticoagulant cascade, such as protein C and protein S, tended to have reduced levels at this time. An important point to emphasize is the plasminogen activator inhibitor (PAI) test, which showed a noticeable increase in activity during pregnancy. The constantly changing levels of hormones had a significant impact on the complex changes in blood clotting control during pregnancy. These changes played a crucial role in protecting the body during important events such as bleeding from the placenta, childbirth, and the time after giving birth.

A lot of research has shown that thrombophilic abnormalities could have serious effects on pregnancy, ranging from miscarriages and the death of the fetus to more complicated problems such as intrauterine growth restriction, placental abruption, and calcification. This type of blood disorder has also been linked to serious problems during pregnancy, such as preeclampsia, eclampsia, and hemolysis, elevated liver enzymes, and low platelet count (HELLP) syndrome [12]. Clinical observations have revealed connections between thrombophilic conditions and complex medical situations such as disseminated intravascular coagulation (DIC) syndrome and fetal bradycardia. This helps us understand the various negative effects of thrombolytic dysregulation on gestational health [13,14].

The clinical scenario discussed here highlights the complex interaction of several contributing factors. Two copies of the mutant allele *MTHFR* (Ala222Val), two copies of *PAI-1* (4G/5G), more NK cells than usual (14.7 per 2-13 norm), more cells working (11.5% per 10% norm), embryotoxin (+) that stopped cell growth, and higher levels of thrombocyte-leukocyte aggregates (2.8 per 1.6% norm) are some of these factors. Despite the presence of many thrombophilic indicators, specific therapies at crucial points in time helped the pregnancy reach full term, demonstrating the effectiveness of focused therapy approaches in reducing negative outcomes.

The plasminogen activator inhibitor (PAI) is an important part of pregnancy because it controls many important processes, including the invasion of trophoblasts during the fertilized egg's implantation and the development of the embryo that follows. It also controlled the complex restructuring of spiral uterine arteries in the endometrium. Disruptions or changes in the *PAI-1* gene could have a series of physiological consequences, resulting in reduced oxygen and nutrition delivery to important embryonic tissues. This abnormality could mess up the coordinated production of important immune memory cells, such as B-cells and tumor necrosis factor-alpha (TNF-α). This could cause a number of bad outcomes, such as miscarriage, preeclampsia, and problems with the baby's development [15].

On the other hand, problems with the *MTHFR* gene or high homocysteine levels in the blood have been linked to a number of reproductive issues, such as early ovarian failure, issues with the attachment of a fertilized egg to the uterus, and multiple miscarriages. MTHFR, an important enzyme in the folate metabolic pathway, is involved in many cellular processes, including the creation of neurotransmitters and phospholipids, as well as the methylation of homocysteine and DNA. Recent studies highlighted a significant link between individuals who have two copies of the *MTHFR* C677T gene variation and negative reproductive results. These consequences included reduced egg quality, increased occurrence of embryos with abnormal chromosome numbers, and decreased embryonic survival capacity [16].

During the early stages of pregnancy, decidual NK (dNK) cells became the main type of NK cells in the uterine milieu. Documents clearly describe the increase in uterine NK (uNK) cells during the secretory phase of the menstrual cycle and early pregnancy, underscoring their critical role in coordinating pregnancy development. Decidual NK cells help blood vessel growth by making ligands that connect with key NK receptor activators. This makes it easier for trophoblasts to move to the endometrial bed. This interaction led to the production of important substances that aid in the growth of blood vessels. These include vascular endothelial growth factor (VEGF) and stromal cell-derived factor-1 (SDF-1). These substances then make the steps for blood vessel formation easier. NK cells had a complex role throughout pregnancy and had a major impact on pregnancy-related disorders, including preeclampsia, recurrent pregnancy loss, and early-stage spontaneous miscarriages [17].

females who experience recurrent pregnancy loss and numerous pregnancy-related illnesses often have embryotoxic substances in their serum. These agents, consisting of a combination of antigens and cytokines, contribute to the development of pregnancy-associated problems. Interestingly, individuals diagnosed with illnesses such as endometriosis and unexplained infertility often exhibit a higher occurrence of embryotoxicity. Studies have shown that pharmacological treatments, such as hormone regulation and immune system modulation, effectively reduce the harmful substances in the mother's blood. As a result, these treatment methods had the potential to create a favorable environment for effective pregnancy development and completion [18].

## Conclusions

The study carefully described the changes that occur in the blood clotting and clot-dissolving systems during pregnancy, revealing the increased risk of blood clot formation in pregnant females. Genetic factors, particularly changes in genes such as *MTHFR* and *PAI-1*, play a significant role in determining pregnancy outcomes such as miscarriages, preeclampsia, and repeated pregnancy loss.

The clinical case studies also gave detailed explanations of tough situations where certain treatments, such as immunomodulatory drugs and anticoagulant regimens, led to good obstetric outcomes despite serious medical conditions. The analysis of elements that cause harm to embryos and the study of the changes in NK cells in the uterine lining have offered extremely significant knowledge about the underlying causes of repeated pregnancy loss and negative outcomes throughout pregnancy.
